# Bacteria from the skin of amphibians promote growth of *Arabidopsis thaliana* and *Solanum lycopersicum* by modifying hormone-related transcriptome response

**DOI:** 10.1007/s11103-024-01444-x

**Published:** 2024-04-14

**Authors:** Yordan J. Romero-Contreras, Francisco González-Serrano, Elena Bello-López, Damien Formey, Wendy Aragón, Miguel Ángel Cevallos, Eria A. Rebollar, Mario Serrano

**Affiliations:** 1 Centro de Ciencias Genómicas, Universidad Nacional Autónoma de México, Cuernavaca, Mexico; 2 Programa de Doctorado en Ciencias Biomédicas, Centro de Ciencias Genómicas, Universidad Nacional Autónoma de México, Cuernavaca, Mexico; 3 Instituto de Biociencias, Universidad Autónoma de Chiapas, Blvd. Príncipe Akishino s/n, 30798 Tapachula, Chiapas Mexico

**Keywords:** *Arabidopsis thaliana*, Biostimulants, Frog skin microbiota, *Solanum lycopersicum*

## Abstract

Plants and microorganisms establish beneficial associations that can improve their development and growth. Recently, it has been demonstrated that bacteria isolated from the skin of amphibians can contribute to plant growth and defense. However, the molecular mechanisms involved in the beneficial effect for the host are still unclear. In this work, we explored whether bacteria isolated from three tropical frogs species can contribute to plant growth. After a wide screening, we identified three bacterial strains with high biostimulant potential, capable of modifying the root structure of *Arabidopsis thaliana* plants. In addition, applying individual bacterial cultures to *Solanum lycopersicum* plants induced an increase in their growth. To understand the effect that these microorganisms have over the host plant, we analysed the transcriptomic profile of *A. thaliana* during the interaction with the C32I bacterium, demonstrating that the presence of the bacteria elicits a transcriptional response associated to plant hormone biosynthesis. Our results show that amphibian skin bacteria can function as biostimulants to improve agricultural crops growth and development by modifying the plant transcriptomic responses.

**Supplementary Information:**

The online version contains supplementary material available at 10.1007/s11103-024-01444-x.

## Key message

Three species of bacteria isolated from amphibian skin exhibited an effect on plant growth in *A. thaliana* and tomato plants. Transcriptomic analysis revealed a transcriptional regulation of hormonal pathways involved in the growth of the plant *A. thaliana*.

## Introduction

For years, chemical fertilizers have been used to change the physical, biological, and chemical properties of soils, which favors the nutritional status of plants, providing the necessary components to improve their development and growth (Neina 2019; Sharma and Chetani 2017). However, excessive application of these chemical agents leads to water, air, and soil pollution, as well as biological imbalances and reduced biodiversity (Kumar et al. 2019; O’Donnell et al. 2001). The use of microorganisms present in the environment has been proposed as an ecological alternative to help promoting plant growth and development, tolerance to biotic and abiotic stress, as well as helping in the assimilation of nutrients stored in the soil or rhizosphere (Ferreira et al. 2019; O’Brien 2017; Rouphael and Colla 2020).

Bacteria that promote plant growth are known as Plant-Growth Promoting Bacteria [PGPB] or Bacteria Plant Biostimulants (De Zelicourt et al. 2013). However, the mechanisms by which PGPBs act on plant biology are still not fully understood in terms of ecology and molecular function (Vandenkoornhuyse et al. 2015). It is proposed that biostimulants function through direct and indirect mechanisms. Direct mechanisms include biological nitrogen fixation [BNF], solubilization of nutrients, such as phosphate, zinc, and potassium (Morales-Cedeño et al. 2021), and secretion of plant growth-promoting substances, including several hormones such as auxins [indole-3-acetic acid or IAA], cytokinins [CK], gibberellins (Basu et al. 2021; Cano 2011; Rehman et al. 2020; Santner et al. 2009; Vega-Celedón et al. 2016), brassinosteroids [BR] (Hussain et al. 2020), and ethylene [ET] (Iqbal et al. 2017). While indirect mechanisms include the production of siderophores that help to solubilize iron [Fe^3+^], and the production of microbial volatile organic compounds [mVOCs] that trigger the induced or acquired systemic defense responses, to combat pathogenic microorganisms (del Rosario Cappellari et al. 2019; Singh 2018).

Biostimulants possess several of the above-mentioned characteristics, which in many cases will depend on changes in the environment where the bacterium develops (Morales-Cedeño et al. 2021). Bacteria of the genus *Azospirrillum* (de-Bashan et al. 2012), *Bacillus* (Sansinenea 2019), *Pseudomonas* (Santoyo et al. 2012), *Burkholderia* (Suárez-Moreno et al. 2012) and *Enterobacter* (Jha et al. 2011), have been reported as plant growth promoters, and/or biocontrol agents [BCAs]. However, many of these microorganisms are often pathogenic to humans, thus posing an ecological and human health risk, which must be addressed before commercial production; furthermore, these PGPBs may also exhibit resistance to plant pathogens (Ramakrishna et al. 2019). Therefore, the identification of new biostimulants and description of the molecular mechanisms behind their beneficial/protective effect are important.

Biostimulant bacteria have been identified from several sources in environment. One promising source is the amphibian skin microbiota since it has been shown that some of their members are able to protect amphibians against fungal diseases such as chytridiomycosis, caused by the chytrid fungus *Batrachochytrium dendrobatidis* [Bd] (Harris et al. 2009; Rebollar et al. 2019). For instance, Susilawati et al. (2021) found that bacteria present on the skin of wild frogs have the potential to control diseases caused by pathogenic fungi in plants. They identified 106 bacteria isolated from three different frogs species [*Hyla japonica*, *Pelophylax porosus porosus* and *Buergeria burger*], three of which significantly inhibited the growth of the fungal pathogen *Colletotrichum orbiculare*, [anthracnose], and produced changes in the root structure of cucumber [*Cucumus sativus*] plants. The molecular mechanisms used by these bacteria to inhibit the plant pathogen and influence plant growth, and development are still undescribed. Moreover, we have recently identified that, bacteria from the genus *Acinetobacter* isolated from the skin of a tropical frog species, reduced the pathogenic activity against *Bd*, and the plant fungal pathogen *Botrytis cinerea* [Bc] (Cevallos et al. 2022). However, their phytostimulant activity in plants is still undescribed.

In order to find bacterial candidates with biostimulant potential, we analysed the growth promoting effect of three bacterial strains isolated from the frog * Craugastor fitzingeri* that inhibit the growth of the fungal pathogens *Bd* and *Bc* (Cevallos et al. 2022; Rebollar et al. 2019; Rebollar and Harris 2019). We determined that growth of *Arabidopsis thaliana* and *Solanum lycopersicum* was improved by the exogenous application of these bacteria. Additionally, to understand the biostimulants molecular effect of one strain [*Acinetobacter* sp. *C32I*], we studied the transcriptomic changes induced by its application on *A. thaliana*, observing modifications in the expression levels for genes related to hormones. These results demonstrate that bacteria from amphibian skin are a good source of bacteria that can have plant biostimulant effects.

## Materials and methods

### Bacteria strain

The bacteria strains used in this study were previously isolated from the skin of the frog *Craugastor fitzigeri* and described in Rebollar et al. (2019). Two of the bacteria were recently identified as members of the *Acinetobacter* genus [C26G and C32I] (Cevallos et al. 2022), and one was only identified at the family level as Enterobacteraceae [C23F] using 16S rRNA sequencing (Rebollar et al. 2019). All of them were able to inhibit the growth of the amphibian fungal pathogen *Bd* (Cevallos et al. 2022; Rebollar et al. 2019)*.* To analyze their effect in the plant, bacteria were cultured on Luria–Bertani medium [LB] and incubated at 30 °C for 24 h until an optical density of 0.6 [O.D. 600 nm] was reached.

### Plant-bacteria interaction in vitro

*Arabidopsis thaliana* ecotype Col-0 seeds were surface sterilized with ethanol 70% three times for five minutes followed by absolute ethanol for ten minutes, the ethanol was decanted and the seeds were placed in square plates containing 0.2× MS agar medium pH 5.7, supplemented with sucrose [0.5% w/v] and 1.5% [w/v] agar (Murashige and Skoog 1962). The plates were placed at 4 °C for 48 h for vernalization and were incubated at 22 ± 2 °C in a plant growth chamber with 16 h/ 8 h light/dark cycles. After four days of germination, 30 µl of each bacterial suspension normalized to an optical density of 0.6 [O.D. 600 nm] was placed on the opposite side of the plants in the plates. Plants were monitored for fifteen days to evaluate primary root growth. At the end of the experiment, root and rosette fresh weight were evaluated. The number of root hairs was analysed under the optical microscope [Zeiss Axioskop 2, 10×] as previously described (Napsucialy-Mendivil and Dubrovsky 2018). The experiments were repeated with at least three biological replicates, each with three technical replicates [5 plants per treatment].

### Histochemical analysis of *A. thaliana DR5::GUS *reporter line inoculated with *Acinetobacter* sp.* C32I*

Sterile seeds of the *A. thaliana DR5::GUS* reporter line were germinated on plates containing 0.2× MS medium pH 5.7. After four days, plates were inoculated with *Acinetobacter* sp. *C32I* bacteria cell suspension. Seven days-post-inoculation [dpi], the seedlings were subjected to GUS histochemical staining during 12 h of incubation at 37 °C in a GUS reaction buffer [0.5 mg/ml of 5-bromo-4-chloro-3-indolyl-β-D-glucuronide in 100 mM sodium phosphate, pH 7] (Jefferson et al. 1987). Tissue clarification was carried out with a solution of methanol: acetone [3:1] during 2 h. For analysis, 20 plantlets [10 plantlets per plate] were analysed at 10 × magnification under a Zeiss Axioskop 2 microscope. Imagens are representative of the experiment. All experiments were carried in triplicate.

### Root hair analyses of the *A. thaliana rdh6* mutant in interaction with *Acinetobacter *spp. *C32I*

For the study, seeds of the *A. thaliana rdh6* mutant, which shows reduced number of root hairs in absence of auxins, were used. Sterile seeds were germinated on plates with MS medium 0.2× pH 5.7. Four-day-old seedlings were inoculated with 30 µl of a suspension of *Acinetobacter* sp. *C32I* cells. After fifteen days of interaction, the seedlings were placed in a 50% sterile glycerol solution for microscopic observation. The number of root hairs was counted as previously described (Napsucialy-Mendivil and Dubrovsky 2018). As control, we used uninoculated seedlings. Ten seedlings were measured for each treatment [n = 10]. All experiments were performed at least three times.

### RNA extraction and leaves transcriptomic analysis of *A. thaliana leaves*

For RNA-seq analysis, *Arabidopsis thaliana* Col-0 plants were root-inoculated with *Acinetobacter* sp. *C32I* under greenhouse conditions*.* Ten leaves per rosette were collected after four weeks post interaction and under uninoculated conditions. Total RNA for RNA-seq was isolated from two different biological replicates for each treatment using the TRizol method according to the manufacturer’s instructions [Invitrogen]. Total RNA concentration and purity were measured with a NanoDrop spectrophotometer [Implen NP80, Thermo Fisher Scientific, USA]. Library construction and sequencing were performed by Beijing Genomics Institute [BGI] Americas2 using DNBSeq TM technology. Differentially expressed genes [DEGs] were identified using the software DESeq2 in the *Integrated Differential Expression Analysis MultiEXperiment* [IDEAMEX] (Jiménez-Jacinto et al. 2019), with a FoldChange ≥ 2, and adjusted *p*-value ≤ 0.05. DEGs were functionally annotated with Gene Ontology terms by PANTHER [v17.0]. GO Term Enrichment for plant analysis was performed by using the web tool TAIR [The Arabidopsis Information Resource [TAIR], https://www.arabidopsis.org/tools/go_term_enrichment.jsp, on www.arabidopsis.org, Feb,24, 2022] employing Fisher’s exact test and correction with an FDR. Non-redundant enriched terms were obtained by using REVIGO software (Supek et al. 2011). Plots were created with the ggplot2 library using R [v4.2.1] libraries ggplot2 and heatmap. KEGG pathways maps were generated by using KEGG Mapper – Color [https://www.genome.jp/kegg/mapper/color.html, Feb, 24, 2022].

### Bacteria- *Solanum lycopersicum* interactions

*Solanum lycopersicum* seeds were placed in 50 mL Falcon tubes and washed three times with 3% sodium hypochlorite and a final wash with absolute ethanol. Seeds were germinated in hydrated vermiculite. Seven-day-old plants were placed in plastic containers containing a mixture of soil and vermiculite [3:1] and maintained under greenhouse conditions with 2 weekly irrigations of 200 mL of tap water. For five weeks, a suspension of bacteria, grown in 0.2× MS medium pH 5.7, was added to the soil every third day. After this time, root and stem length, fresh, dry were measured. We performed two biological replicates, each with 8 plants per treatment. The experiment was replicated to assess both production and fresh weight.

### Statistical analysis

All results are reported as mean values [± SEM]. A one-way ANOVA for non-parametric data and Tukey´s analysis [p < 0.05] was carried out to determine statistically significant differences between each experiment. The software GraphPad Prism version 9.2.0 was used [GraphPad Software, San Diego, CA, USA, 2019]. All the data analysed were obtained from three independent experiments.

## Results

### Frog skin microbiota modify the root structure of *A. thaliana*

**Fig. 1:**
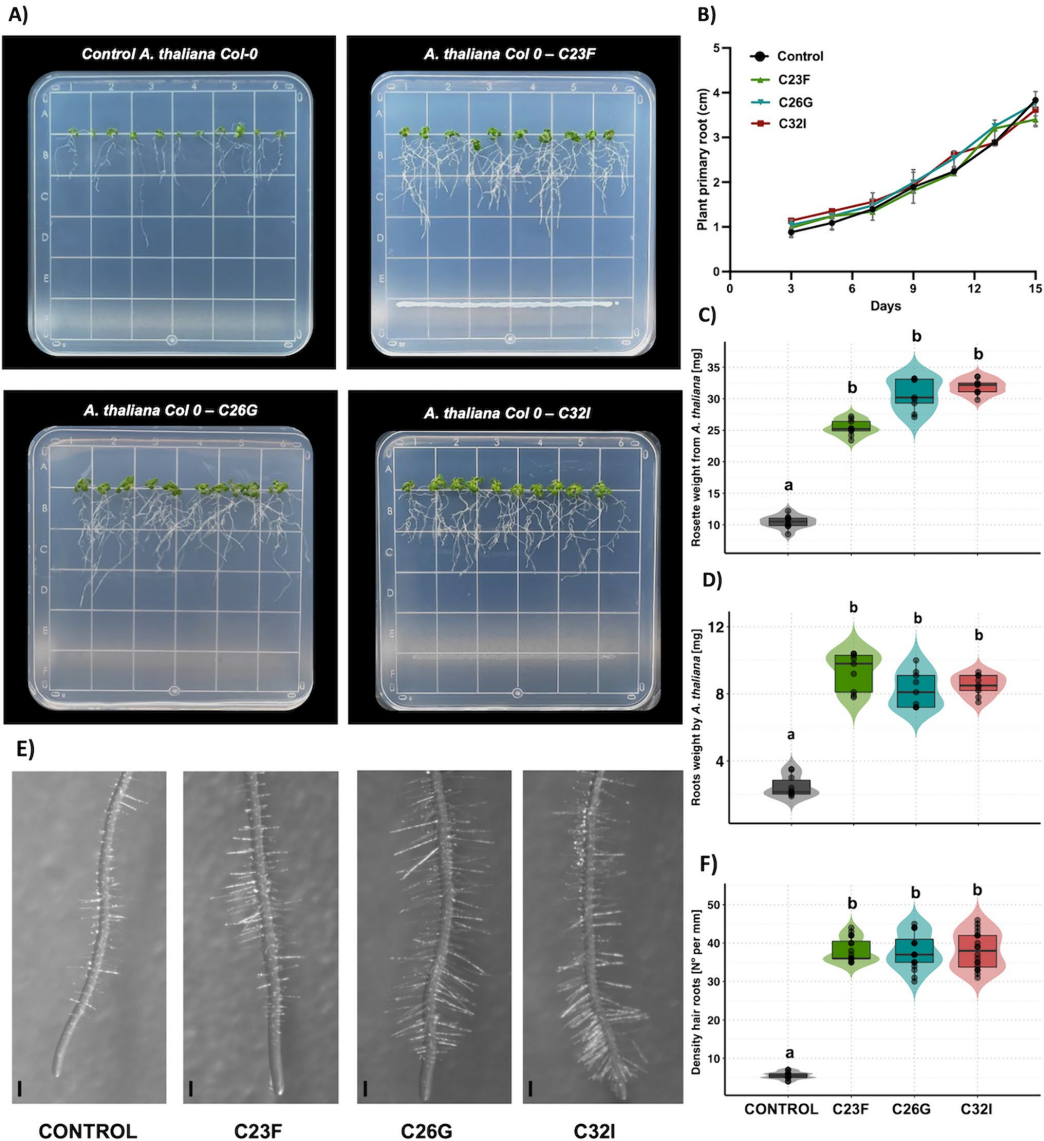
Frog skin bacterial isolates modify the root structure of *A. thaliana*. Four-day-old *Arabidopsis thaliana Col-0* seedlings were exposed, individually, to 30 µl of cell suspensions containing C23F, C26G, or C32I bacteria. Plant growth was observed over a 15 dpi. We conducted an analysis to evaluate the influence of each bacterial strain on root hair production in *Arabidopsis* roots. (**A**) Representative images of the interaction of each bacterium with the plant, with uninoculated plants using as the control. (**B**) Root length was monitored throughout the experiment from the apical zone to the stem collar. (**C**) Fresh weight of rosettes and (**D**) fresh weight of roots in *A. thaliana* plants were determined. (**E**) Frog skin bacteria were found to modify the root structure, leading to an increase in the number of root hairs in the apical zone. (**F**) Quantification was performed per millimeter of both inoculated and non-inoculated plant roots. Letters indicate a statistically significant difference, according to one-way analysis of variance [ANOVA] [p ≤ 0.05] followed by a Tukey test. Scale bar 100 µm

To investigate the effect of bacteria on the growth of *A. thaliana* Col-0 plants, seeds were germinated on MS 0.2× for four days under in vitro conditions, then inoculated with bacterial isolates C23F, C26G, and C32I, and compared to the uninoculated control. After fifteen days post interaction [dpi], we evaluated the primary root growth, root and stem fresh weight, and the number of root hairs for each treatment (Fig. 1). The results obtained showed that there was no significant difference in the growth of the primary root of *A. thaliana* between the control and the treatments (Fig. 1B). However, when evaluating the fresh weight of both root and stem, we observed that the plants in the presence of each bacterium individually presented a significantly higher weight compared to the uninoculated control (Fig. 1C, D). Additionally, it was observed that all bacteria modified the root structure of *A. thaliana* by promoting the growth of secondary roots and the development of root hairs (Fig. 1E). Our analysis showed that plants inoculated with C23F promoted root hair formation approximately of 40 roots hair/mm, followed by C26G and C32I with 38 and 34 roots hair/mm respectively, compared to the control which showed only five roots hair per mm (Fig. 1F). These data correlate with the increase in root and stem mass, which could confer a benefit on the plant for water and macronutrient and micronutrient uptake.

### Presence of frog skin C32I bacteria induces transcriptional changes in the *A. thaliana* plant

**Fig. 2:**
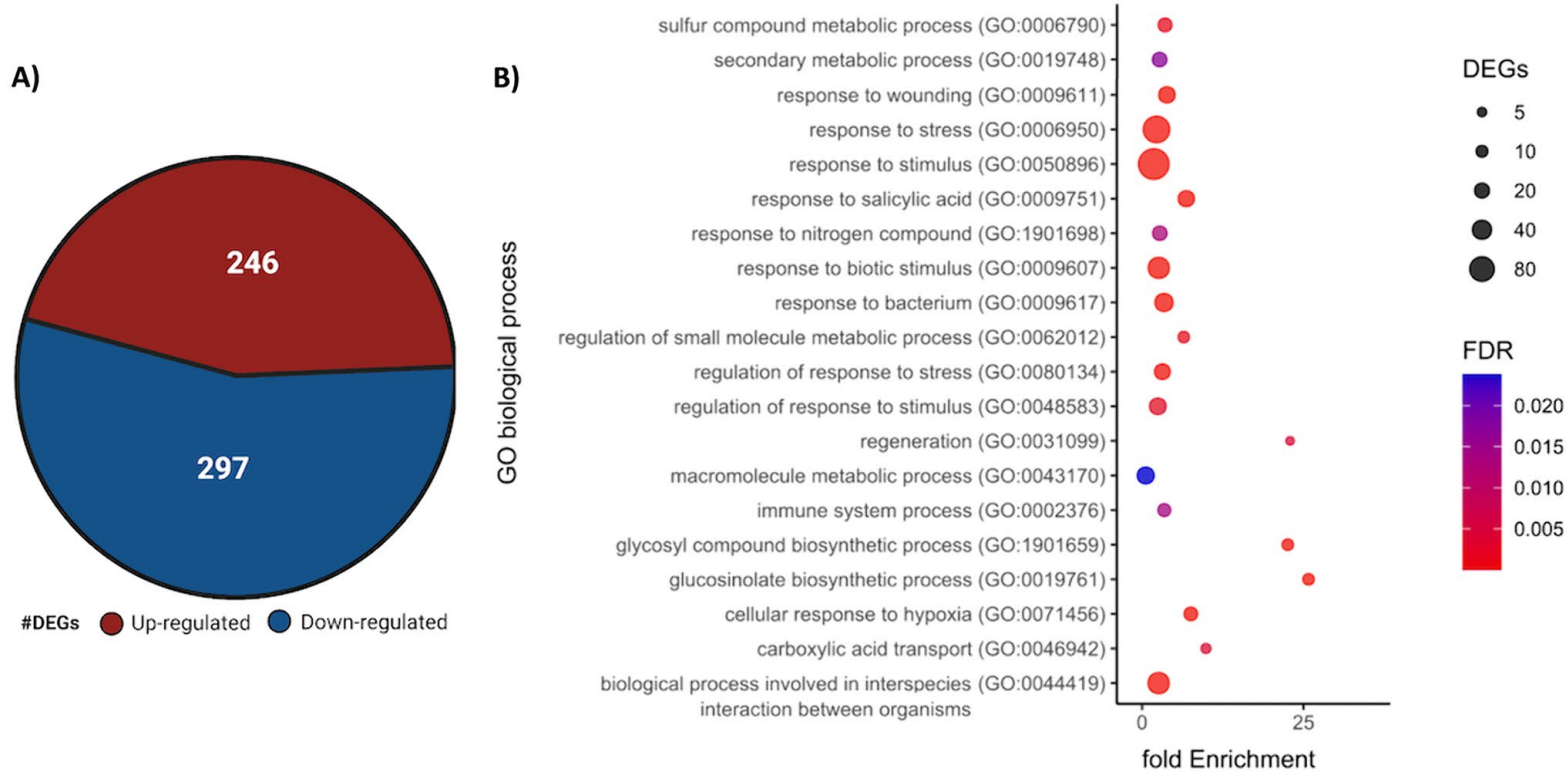
Transcriptome analysis of *A. thaliana* leaves in response to *Acinetobacter* spp. C32I*.* Plants of *A. thaliana Col-0* were inoculated with the cellular suspension of *Acinetobacter* sp. C32I at the root level. Leaves-collected from each plant for sequencing [RNA-seq] of total RNA. (**A**) Venn diagrams show the number of up-regulated and down-regulated DEGs identified in the treatment versus the control [Log2FC ≥ 2 or ≤ -2]. (**B**) Distribution of the different biological functions of Gene Ontology [GO] for the up-regulated genes [*p* < 0.05]. The circle size represents the number of genes biological process associated. The graph represents the enrichment of “Biological process” GO-terms [FDR < 0.01]

To elucidate the molecular effect of frog skin bacteria on growth of *A. thaliana*, we selected the C32I bacteria because it generated the greatest change in plant growth under *in-vitro* conditions (Fig. 1). After four weeks we collected rosette-leaves samples from uninoculated and inoculated plants and analysed the expression levels by RNAseq (Supplementary Data 1, Fig. 2). We identified that the number of differentially expressed genes [DEGs] in plants inoculated with C32I were 543 versus the control treatment without bacteria. Two hundred forty-six genes were up-regulated and 297 were down-regulated respectively (Fig. 2A).

To further identify the plant biological processes that changed in response to the presence of C32I, a comparison analysis between uninoculated and inoculated plants was performed using REVIGO software. Our results showed that 543 genes were significantly classified into 20 biological processes. From the up-regulated genes, we identified GO categories for: sulfur compound metabolic process, secondary metabolic processes, response to wounding, response to stress, response to stimulus, response to bacterium, response to biotic stimulus, regeneration, macromolecule metabolic process and biological processes involved in interspecies interaction between organisms, related to the biological activity of the plant (Supplementary Data 2, Fig. 2B). The analysis suggests that plants in the presence of C32I bacteria, triggers the expression of various functional groups of genes that are usually associated with the interaction between plants and bacteria (Kudoyarova et al. 2019), promoting plant growth and defence mechanisms. These results help to explain the observed traits of the plants previously observed in the *in-vitro* analysis.

### *Acinetobacter* sp. C32I induces transcriptional changes on plant hormonal signal transduction pathways

**Fig. 3:**
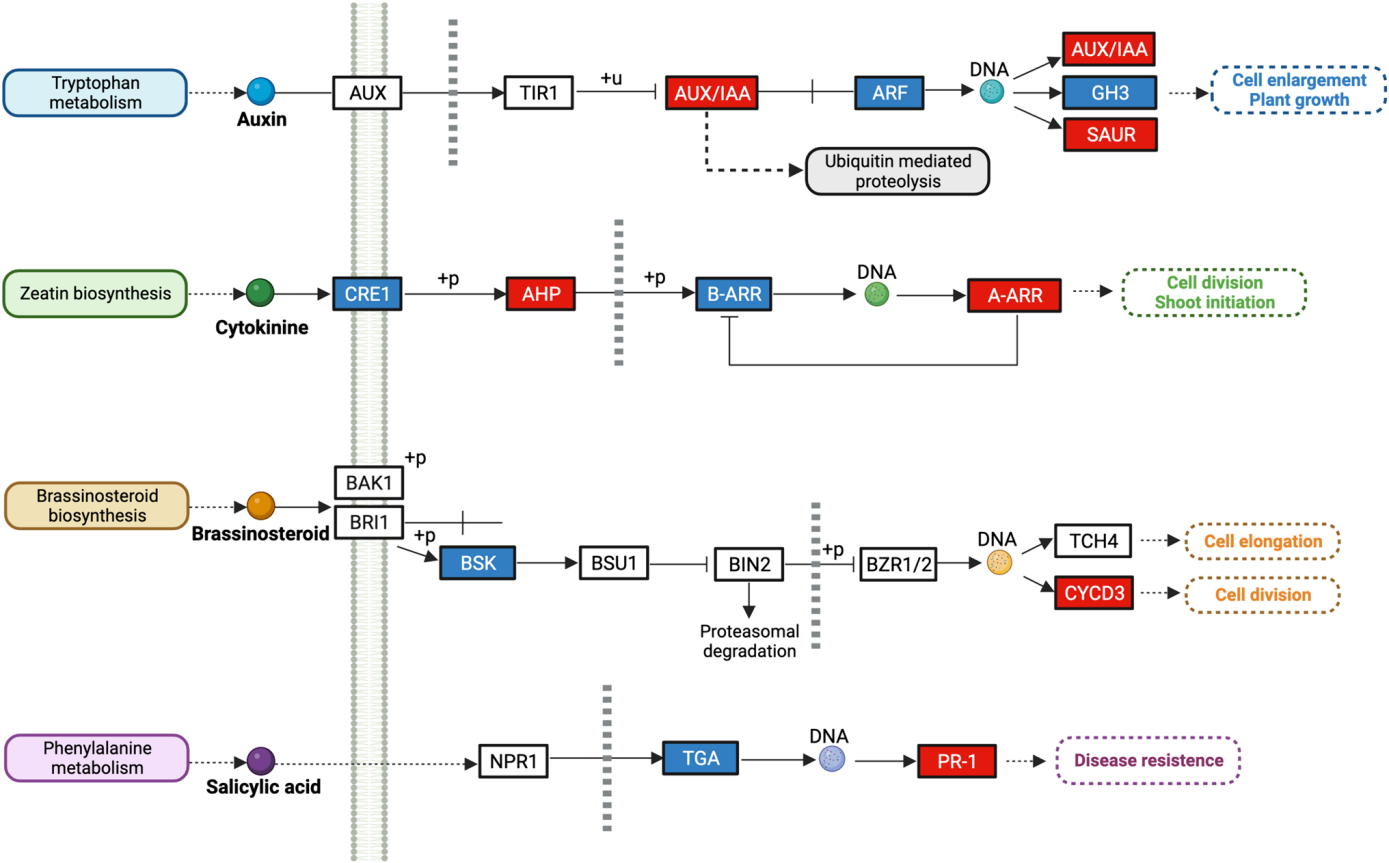
KEGG pathway image of plant hormonal signal transduction DEGs related to plant growth in *A. thaliana* plants treated with *Acinetobacter* sp. C32I. Relative transcript abundance of genes of plant growth-related hormones after four weeks interaction with bacterium compared to the uninoculated control. The DEGs coding for each gene family are represented by boxes. Identified genes related to hormone transduction pathways related to Auxin [AUX], Cytokinin [CK], Brassinosteroid [BR], and Salicylic Acid [SA] synthesis. Red boxes represent up-regulated genes and blue boxes represent down-regulated genes. IAA: indole acetic acid, ARF: auxin response factor, GH3: Gretchen Hagen 3, SAUR: small auxin upregulated, BSK: brassinosteroid-signaling kinase, CYCD3: D-type cyclin, CRE1: cytokinin response 1, AHP: histidine phosphotransfer, B_ARR: Arabidopsis response regulator type B, A_ARR: Arabidopsis response regulator type A, TGA: TGACG-binding factors, PR1: protein pathogenesis-related

Since we found a growth promoting effect of frog skin bacteria on the plant, we investigated the hormone biosynthetic pathways that could induced by the presence of C32I. We found 33 genes from Auxin [AUX], Cytokinin [CK], Brassinosteroids [BR] and Salicylic Acid [SA] biosynthesis pathways using the Kyoto Encyclopedia of Genes and Genomes [KEGG] analysis (Supplementary Fig. 1). With respect to the AUX pathway, 7 genes related to Indole-Acetic Acid [IAA] biosynthesis were identified, of which genes *IAA3*, *IAA27*, *IAA17*, *IAA28*, *IAA19* and *IAA8*, showed an increase in expression in inoculated plants, except for the gene *IAA2* which showed a decrease its expression. Similarly, *ARF1* and *ARF7* were down-regulated in inoculated plants; these genes are transcription factors [TFs] which bind to auxin response elements [AREs], that carefully regulate the plant’s response, and prevent inappropriate overexpression of certain genes in response to auxin (Mallick et al. 2022). On the other hand, it was observed that the genes corresponding to the small auxin up-regulated RNA [SAUR] gene family showed an increase in expression in inoculated versus uninoculated plants, including *SAUR6*, *SAUR3*, *SAUR26*, *SAUR31* and *SAUR14* (Fig. 3, Supplementary Data 3).

Based on the gene expression involved in the auxin biosynthesis, the transcriptomic levels of the genes of the cytokinin [CK] (Kakimoto 2003) and brassinosteroid [BR] (Chung and Choe 2013) pathways were evaluated. We observed that plants inoculated with *Acinetobacte*r sp. C32I showed up-regulation of *CYCD3* gene expression. This gene, involved in the CK pathway, has a crucial function in the regulation of the cell cycle, DNA synthesis, and plant development, while *BSK8* was down-regulated (Fig. 3, Supplementary Data 3). Continuing with cytokinins, the highest expression levels were found in for the AHP family of genes encoding for a histidine phosphotransfer proteins such as *AHP2* and *AHP1*. Interestingly, members of the Response Regulator Gene Family to Cytokinin [ARR gene family] which is subdivided into type A and B (Ferreira and Kieber 2005), were identified where two typo B genes *ARR5* and *ARR7* and three typo A genes *ARR3*, *ARR16*, and *ARR9* were found to be expressed in the plant in response to the presence of the bacteria versus the control (Fig. 3, Supplementary Data 3).

The phytohormone salicylic acid [SA] is one of the best known and described molecules involved in plant biology, specifically during plant-pathogen interactions (Maruri-López et al. 2019). Based on this knowledge, we observed that the expression of *TGA1*, *TGA2*, and *TGA4* were down-regulated while *PR1* was up-regulated. Taken together, the transcriptomic analysis of the interaction of *A. thaliana* plants inoculated with *Acinetobacter* sp. C32I suggest that the bacterium triggers plant development through the modification of induction of phytohormones-mediated signalling pathways.

### *Acinetobacter* sp. C32I induces auxin production and accumulation in the *A. thaliana*

**Fig. 4:**
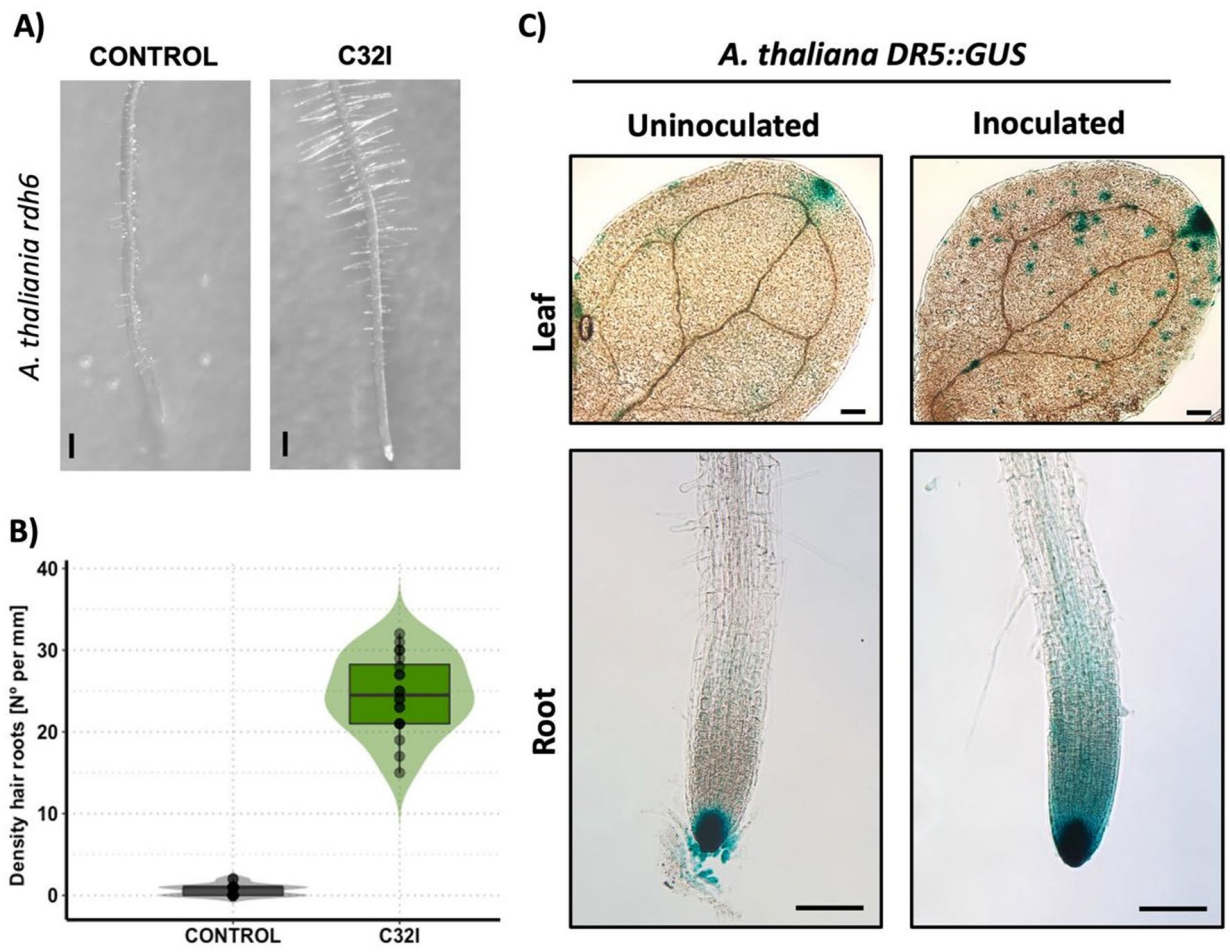
*Acinetobacter* sp. C32I induces auxin production and accumulation. *A. thaliana rdh6* mutant seeds deficiency in root hair production and DR5::GUS reporter line, were germinated on plates containing 0.2× MS medium in the presence of C32I bacterium. The parameters were evaluated at 7 dpi. (**A**) Representative images of each treatment are show. The plant treated with *Acinetobacter* sp. *C32I* exhibits an induction in the production of root hairs, in contrast to the non-inoculated control. (**B**) The analysis of the roots in *rdh6* plants, unveiled an increased count of root hairs in plants inoculated with the bacteria in comparison to the uninoculated control. (**C**) The DR5::GUS reporter line shows a different signal in leaves and roots in the presence of the bacterium versus control. The experiments were repeated three times with similar results [n = 20 ± SD]. Letters indicate a statistically significant difference, according to a one-way analysis of variance [ANOVA] [*p* ≤ 0.05] followed by the Tukey test. Mocks represent the plants with MS medium. Scale bar for *rdh6* 200 µm. Scale for DR5:GUS 100 µm

To validate our transcriptomic analysis, we verified the accumulation and biosynthesis of auxin in *A. thaliana* in the presence of C32I. First, we were interested to know if this bacterium regulates and restores the WT phenotype of the *rdh6* mutant, deficient in auxin-mediated root hair production (Masucci and Schiefelbein 1994). Thus, we inoculated four-day-old plants with the *Acinetobacter* sp. C32I, and then analysed the root structure in plants by microscopy, using uninoculated plants as controls (Fig. 4). We found was that plants inoculated with *Acinetobacter* sp. C32I presented a higher number of root hairs, compared to the control, where there was a deficiency in hair production as expected (Fig. 4A, B). To further investigate the role of C32I in root development, we analysed auxin accumulation and distribution in roots using the *A. thaliana DR5:GUS* reporter line (Ulmasov et al. 1997). For this purpose, four-day-old plants were inoculated with the isolate C32I in direct interaction, and after seven dpi the roots were analysed by microscopy. The histochemical staining pattern of inoculated plants showed a higher GUS signal in the apical zone of the root and the foliar zone of the leaf corresponding to sites of high auxin production, in contrast to the control (Fig. 4C). We conclude that auxin production and accumulation are induced in the presence of the bacteria, and this observation supports by our transcriptomic analysis.

### Frog skin microbiota promote growth of tomato plants

**Fig. 5:**
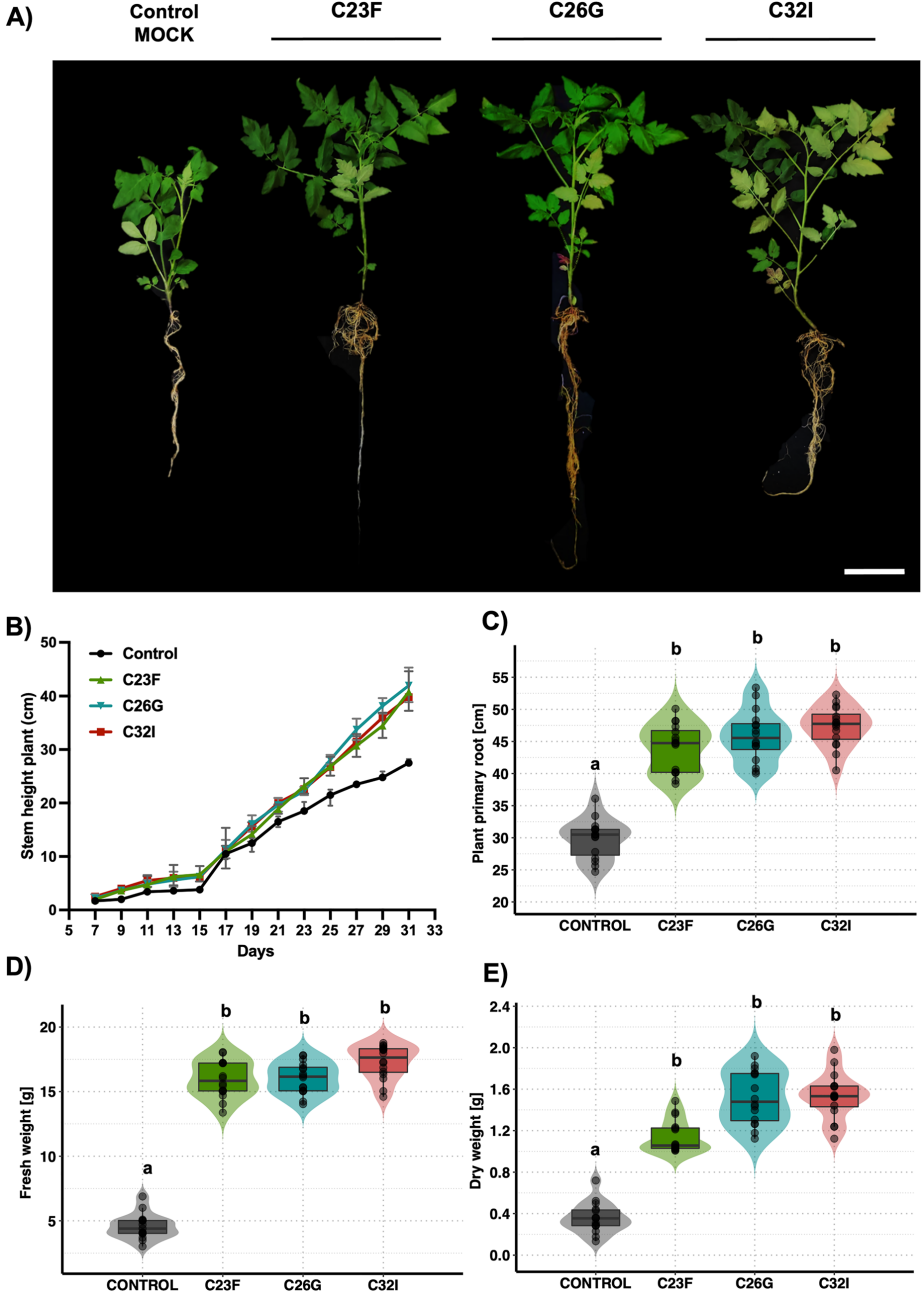
Effect of soil inoculation with frog skin bacteria on the growth of tomato plants. Tomato plants were germinated under greenhouse conditions, periodic inoculated with C32I bacterium. After 5 weeks of interaction, morphological parameters of the plants were evaluated. (**A**) Representative images for each treatment display a increase in plant growth when treated with individual bacteria, as compared to the non-inoculated control. (**B**) Stem length over time. (**C**) Stem length. (**D**) Fresh weight. (**E**) Dry weight. Graphs represent five plants per treatment. Letters indicate a statistically significant difference, according to a one-way analysis of variance [ANOVA] [*p* ≤ 0.05] followed the Tukey test. Mocks represent the plants with MS medium. Scale bar 10 cm

Previously, we found that three bacterial isolates obtained from frog skin induced and modified the growth and root structure of *A. thaliana* (Fig. 1). As part of the characterization of the three isolates, we decided to determine the effect of these bacteria on the economically important crop model tomato [*S. lycopersicum*]*.* First, we monitored the growth of the stem for five weeks, and observed that plants inoculated individually with C23F, C26G, and C32I isolates, after seventeen days, significantly increased in height compared to the control without bacteria (Fig. 5B). Subsequently, the plants were removed from the greenhouse and carefully washed to remove the substrate. Roots were measured and had a greater length in roots inoculated with each bacterium compared to plants without bacteria (Fig. 5C). Biomass was measured [fresh and dry weight], where the plants treated with bacteria showed an increase of 150% compared to control plants (Fig. 5D, E). This analysis suggests that these bacteria from the amphibian skin may exert a beneficial effect on tomato plant development and thereby improve plant performance compared to uninoculated controls.

**Fig. 6:**
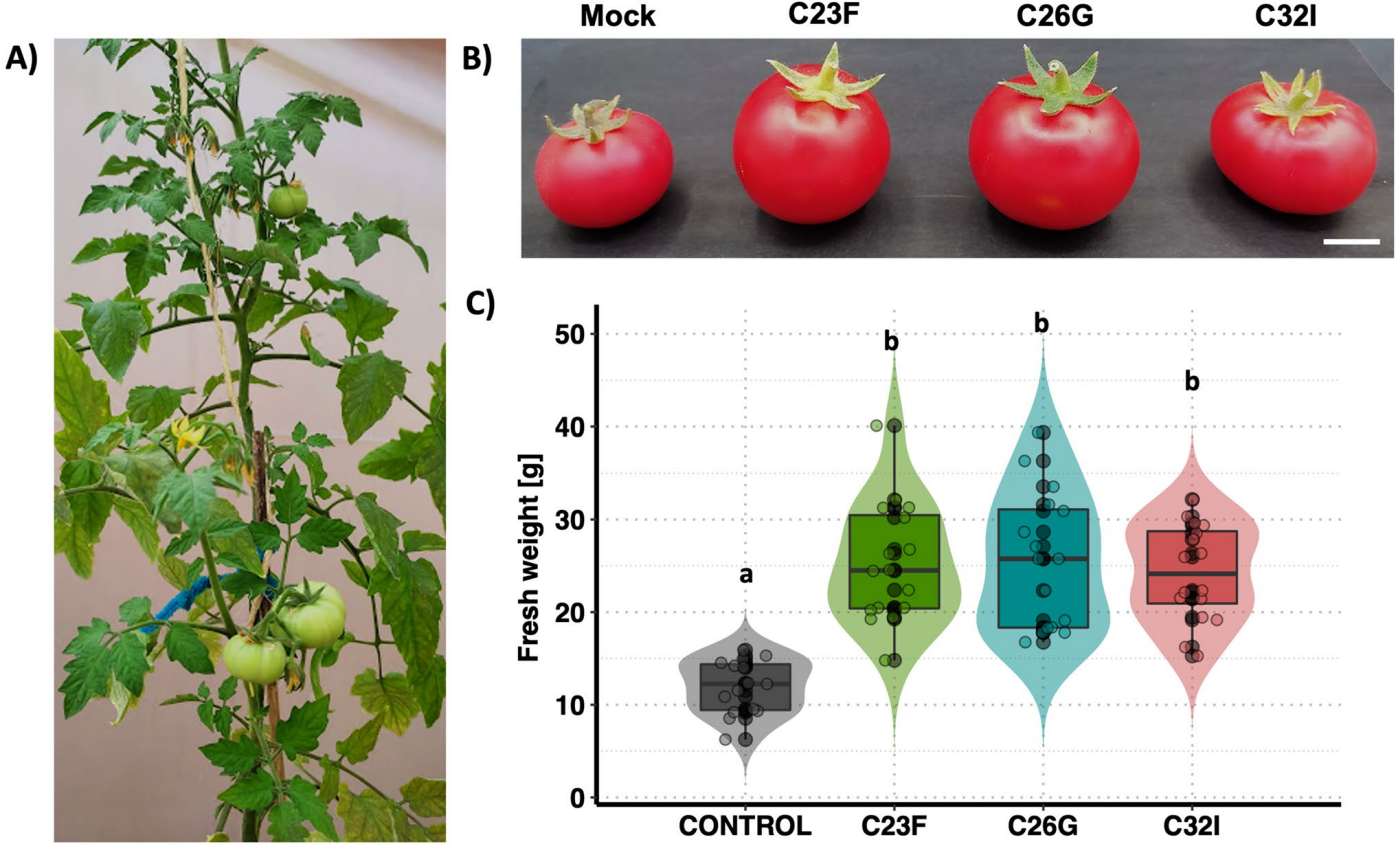
Effect of frog skin bacteria C32I on tomato fruit production. Tomato plants treated with C32I bacterium, were cultivated until that produced mature fruits [four months post inoculation]. (**A**) Representative image of the control treatment. (**B**) In the treatments with each bacterium individually, a larger size is evident compared to the non-inoculated control, where the fruit exhibited a smaller size. Mock indicate inoculation with MS 0.2× medium as a control. (**C**) Quantification of fresh weight of tomato fruits. The graphs represent two experiments that were done [n = 20 ± SD]. Letters indicate a statistically significant difference, according to a one-way analysis of variance [ANOVA] [*p* ≤ 0.05] followed the Tukey test. Scale bar 1 cm

To determine the effect of the bacteria on the size and number of tomato fruits, the plants were maintained for four months in a greenhouse, with periodic inoculation of the bacteria cultures and constant water irrigation (Fig. 6A). The data obtained showed no differences in the number of fruits per plant of each treatment (Supplementary Fig. 2), however, during harvest treated plants with each bacterium showed a larger size of fruits compared to the control without bacteria (Fig. 6B). A measurement of the fruits of each treatment showed that the control had an average of approximately 12 g per fruit, while the inoculated tomatoes showed a biomass of approximately 23–25 g per fruit (Fig. 6C). The results suggest that although there were no differences in the number of fruits, an increase in the size of the tomatoes was observed, which suggests frog skin bacteria can influence the growth of the plant and the size of the fruits.

### Transcriptional reprograming induced by C32I is different than other BCAs previously reported

**Fig. 7:**
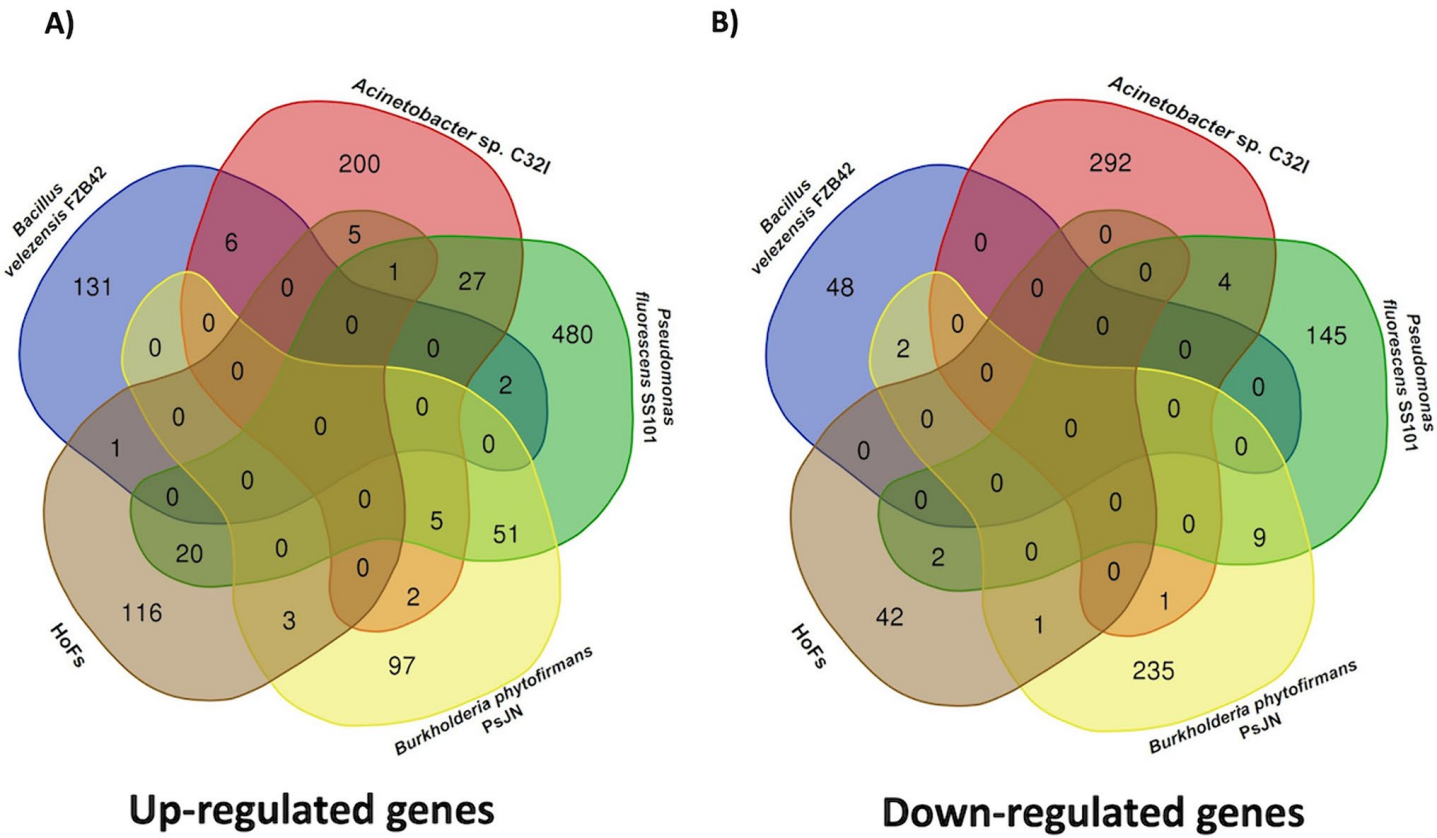
Comparative transcriptomic analysis between other biocontrol agents. Transcriptomic data of *A. thaliana* with *Acinetobacter* sp. C32I, were compared with *Pseudomonas fluorescens SS101*, *Bacillus amyloliquefaciens FZB42*, *Burkholderia phytofirmans PsJN* and *Hanseniaspora opuntia* [HoFs] biocontrols previously reported. Venn diagrams representing overlapping of (**A**) upregulated and (**B**) downregulated genes

Very few reports have analysed the transcriptomic responses of plants stimulated by the presence of PGPBs. In order to compare our results with other biostimulant agents, we compared the differentially expressed genes [DEGs] with transcriptional reports of other bacteria such as *Pseudomonas fluorescens SS101*, *Bacillus amyloliquefaciens FZB42*, *Burkholderia phytofirmans PsJN* (Poupin et al. 2013; Tzipilevich et al. 2021; van de Mortel et al. 2012), and compounds released by the yeast *Hanseniaspora opuntia* [HoFs] (Ferreira-Saab et al. 2018) (Fig. 7, Supplementary Data 4). Interestingly, we found that there is no central core of differentially expressed genes [DEGs] among all the reported agents, indicating that each strain or compound elicits a plant-specific response. However, each bacterium does show a pattern of overexpression in functional groups related to plant growth (Supplementary Data 4). For instance, *SS101* exhibited genes associated with root morphogenesis, secondary metabolism, and SA signalling (van de Mortel et al. 2012). Similarly, previous studies have reported that inoculation of *A. thaliana* with *PsJn* (Poupin et al. 2013) and *FZB42* (Tzipilevich et al. 2021), respectively, led to overexpression of hormone-related pathways, particularly auxins. These findings align with the results of our study, where we observed the up-regulation of genes related to hormone biosynthesis.

## Discussion

Chemical fertilizers and other agrochemicals have been used to promote plant growth, however, it has been observed that these chemicals cause harmful effects on the environment and human health, so environmentally friendly alternatives are needed (Basu et al. 2021). In nature, plants interact with diverse microorganisms such as bacteria, fungi, nematodes and viruses (Compant et al. 2010; Khare et al. 2018). With respect to bacteria, they can establish a beneficial association with plants, favoring plant growth, enhanced defense systems, and tolerance against biotic and abiotic stresses (Custodio et al. 2022; Eichmann et al. 2021; Islam et al. 2020; Jones et al. 2019; Kang et al. 2019; Vandenkoornhuyse et al. 2015; Woodhams et al. 2023). Similarly, amphibians such as frogs and salamanders are known to possess a microbial diversity in their skin, which protects them against various pathogens such as chytrid fungus *B. dendrobatids* a pathogen responsible for the extinction of several amphibian species (Rebollar et al. 2018; Scheele et al. 2019; Varga et al. 2019). Whilst several microorganisms isolated from soil that have been characterized as biostimulant agents, bacteria from the amphibian skin has recently gained attention as many of them have antifungal properties and could likely promote plant growth (Rebollar et al. 2020; Woodhams et al. 2015).

On the other hand, bacteria associated with both plants and amphibians can indirectly influence each other through their biotic and abiotic interactions (Berendsen et al. 2012; Rebollar and Harris 2019). It has been proposed that changes in the diversity of bacteria present in animals and plants may be influenced by variations in precipitation, thereby facilitating the exchange of microbial communities between habitats (Bernardo-Cravo et al. 2020; Ikeda-Ohtsubo et al. 2018; Van Stan II et al. 2020). Despite the evidence of bacteria-host interactions in plants or animals, and on the antifungal mechanisms exerted on pathogenic fungi, it is not known whether bacteria isolated from amphibian skin can exert beneficial effects on plant growth. To our knowledge, there is only one report in which bacteria from three different amphibian species were characterized as biostimulant plant growth modifying agents. This study included 106 bacteria, of which three were selected to study their interaction with cucumber [*Cucumus sativus*] plants. These three bacteria, identified as HjD57, HjD92 [isolated from *Hyla japonica*], and B341 [isolated from *Buergeria burgeri*], did not produce changes in germination or shoot production and only B341 produced changes in root growth (Susilawati et al. 2021). Our study shows that three bacterial strains isolated from tropical frog *Craugastor fitzigeri* (Rebollar et al. 2019) named C23F, C26G, and C32I did produce changes in the growth of the *A. thaliana* model plant, mainly in the roots. In particular, those belonging to the genus *Acinetobacter* (Cevallos et al. 2022), are of particular interest, since bacteria from this genus had previously shown to promote plant growth and development (Molina-Romero et al. 2017).

Moreover, to characterize the molecular effects underlying *A. thaliana*, we selected one of the previously characterized bacteria. We identified that *Acinetobacter* sp. C32I regulates several biological processes related to plant growth and development. Interestingly, a previously study found that tobacco plants [*Nicotiana tabacum*] inoculated with the bacterium *Bacillus cereus*, showed significant differential expressions in categories related to biological processes, mainly in plant hormone signal transduction (Li et al. 2020). Our KEGG analysis identified genes [DEGs] that are up-regulated from the auxin, cytokinin, brassinosteroid, and salicylic acid pathways. Multiple reports have implicated the direct involved of these phytohormones in the modification of plant leaf and root structure (Spaepen 2014) (Fig. 3). Additionally, these results are similar to previous studies, where microorganisms such as *Pseudomonas, Agrobacterium, Rhizobium, Bradyrhizobium* and *Azospirillum*, can regulate the biosynthesis of hormones, specifically auxins (Costacurta and Vanderleyden 1995).

Auxins are one of the most important hormones in plant life, and it is known that soil-borne bacteria produce indole-3-acetic acid [IAA], whose main function is to contribute to plant root growth (Zhao 2010). Spaepen et al. (2014) reported that the bacterium *Azospirillum brasilense* contributes to the increase of lateral roots and root hairs, increasing the concentration of internal auxin in the plant. Similarly, Samaras et al. (2022) reported that inoculation of the *Bacillus subtilis MBI600* in cucumber [*Cucumis sativus L*.] altered the expression of genes involved in phytohormone production, mainly in indole-3-acetic acid-induced genes *ARG7* and auxin-responsive proteins Csa_2G011420 and Csa_3G035310. Interestingly, in our study *A. thaliana* showed an increase in the expression of auxin hormone-related genes after inoculation with C32I, mainly those related to structural changes in the root. Similarly, Lakshmanan et al. (2013) also described that *A. thaliana* inoculated with *Bacillus subtilis FB17* showed significant changes in auxin-responsive genes *AT1G29460* and *AT1G29500*. Collectively, our study indicates that the amphibian skin bacteria are involved in auxin-mediated root development reprograming. Therefore, it is likely that this microorganism acts beneficially in modifying growth and plant development supporting the proof of concept that implementation of microorganisms from non-plant systems could be implemented as new ecological alternatives that act in benefit of the plant.

On the other hand, auxin can interact with other hormones like ethylene, collectively contributing to root development by inhibiting main root elongation and promoting root hair emergence (Hu et al. 2017; Weijers et al. 2018). Furthermore, soil microbial communities can influence auxin concentrations (Compant et al. 2019). Studies show that *Pseudomonas *sp. SP01 affects auxin distribution (Chu et al. 2020). In another study, Méndez-Gómez et al. (2021) report that indirect contact of *A. thaliana* DR5:GUS plants with *Azospirillum brasilense* reduces the GUS signaling, while direct contact increases the signal. Our study supports these findings, demonstrating that direct inoculation of *Acinetobacter* sp. C32I influences auxin accumulation, particularly in the root apical zone. However, further research is needed to understand auxin biogenesis and distribution during plant morphogenesis.

To look deeper into the effect that biostimulant agents have in growth and development, mutant plants like *A. thaliana rdh6* [ROOT HAIR DEFECTIVE 6] have been used. Previous studies have demonstrated that *A. brasilense Sp245*, induces lateral roots and root hairs in *rdh6* mutants (Spaepen et al. 2014). These results are like ours, in which C32I reestablished the production of root hairs compared to the control. Our study suggests that components of auxin and ethylene [signalling and transport] play a crucial role during beneficial microbe interactions. Collectively, our findings indicates that amphibian skin bacteria can induce auxin-mediated root development reprograming, thus promoting plant growth and plant development. This supports the proof of concept that implementation of microorganisms from non-plant systems could be implemented as new ecological alternatives that act in benefit of the plant.

There are several studies that have demonstrated the influence of bacteria on crop improvement and productivity, mainly in the acquisition of nutrients, fixation and solubilization of insoluble minerals, production of siderophores, regulation and production of phytohormones and improvement of the plant defense system (Hamid et al. 2021). Indeed, there are several bacteria that have been characterized as biostimulants in crops such as the *Bacillus* spp. (Sansinenea 2019), *Pseudomonas* spp. (Widnyana and Javandira 2016), *Burkholderia *spp. (Coutinho et al. 2015), *Acinetobacter* spp. (Molina-Romero et al. 2017; Mujumdar et al. 2023), and others. Additionally, it has been proposed that the mixture of different bacterial species through a consortium can enhance plant development (Molina-Romero et al. 2021). Application of bacteria from the skin of amphibians to rice and cucumber, showed a protective effect against pathogens but did not improve their growth and development (Susilawati et al. 2021). We demonstrate the effect of amphibian skin bacteria on the growth and development of the *A. thaliana*, along with transcriptional changes elicited in the host after bacterial inoculation.

To determine if the effect of this bacterium could be similar in an agronomical important crop. We showed that C23F, C26G and C32I improved growth of tomato plants [*Solanum lycopersicum*], demonstrating the potential of amphibian skin bacteria to improve plant growth and development in several plant species. Therefore, the discovery of new biostimulant agents can be of great help to replace chemical agents that affect the environment. However, we still need studies to prove the potential of these bacteria in agricultural soils and their implication as a new ecological alternative and that are harmless to human health.

## Conclusion

Plant growth-promoting bacteria have the potential to be used as biostimulants for the benefit of different crops of agricultural interest. However, the potential use of bacteria from animals such as amphibians as new and efficient agricultural inoculants has been poorly explored. We demonstrate, in this study, that three bacteria isolated from the skin of frogs, two *Acinetobacter *sp. C26G and C32I, and one *Enterobacteraceae* C23F, improve plant development in *A. thaliana* plants mainly in the modification of root structure, production of a greater number of root hairs and an increase in biomass. Additionally, we identified that inoculation with one of these bacteria [C32I], triggers transcriptomic changes in *A. thaliana*, inducing the expression of genes related to plant growth hormones, specifically auxins. Finally, we identify that the inoculation of the three bacterial isolates in a plant of agricultural interest, such as tomato, produces an increase in the length of the root and stem, and an increase in the biomass of the fruits. Based on the results, our study can contribute to the identification and characterization of potential PGPBs from animals to improve the development and growth of agronomically important plants.

### Supplementary Information

Below is the link to the electronic supplementary material.

Supplementary file1 (XLSX 1020 KB)

Supplementary file2 (XLSX 11 KB)

Supplementary file3 (XLSX 16 KB)

Supplementary file4 (XLSX 45 KB)

Supplementary file5 (TIFF 217 KB)

Supplementary file6 (TIFF 12155 KB)

## Data Availability

The RNA sequencing data produced in this study have been submitted to the NCBI’s Sequence Read Archive and are accessible under the following link: https://www.ncbi.nlm.nih.gov/bioproject/PRJNA986187
