# Supplementary figures and images for: Bacteria from the skin of amphibians promote growth of Arabidopsis thaliana and Solanum lycopersicum by modifying hormone-related transcriptome response
Source: Plant Mol Biol. 2024 Apr 14;114(3):39. doi: 10.1007/s11103-024-01444-x (PMC11016013; doi:10.1007/s11103-024-01444-x)

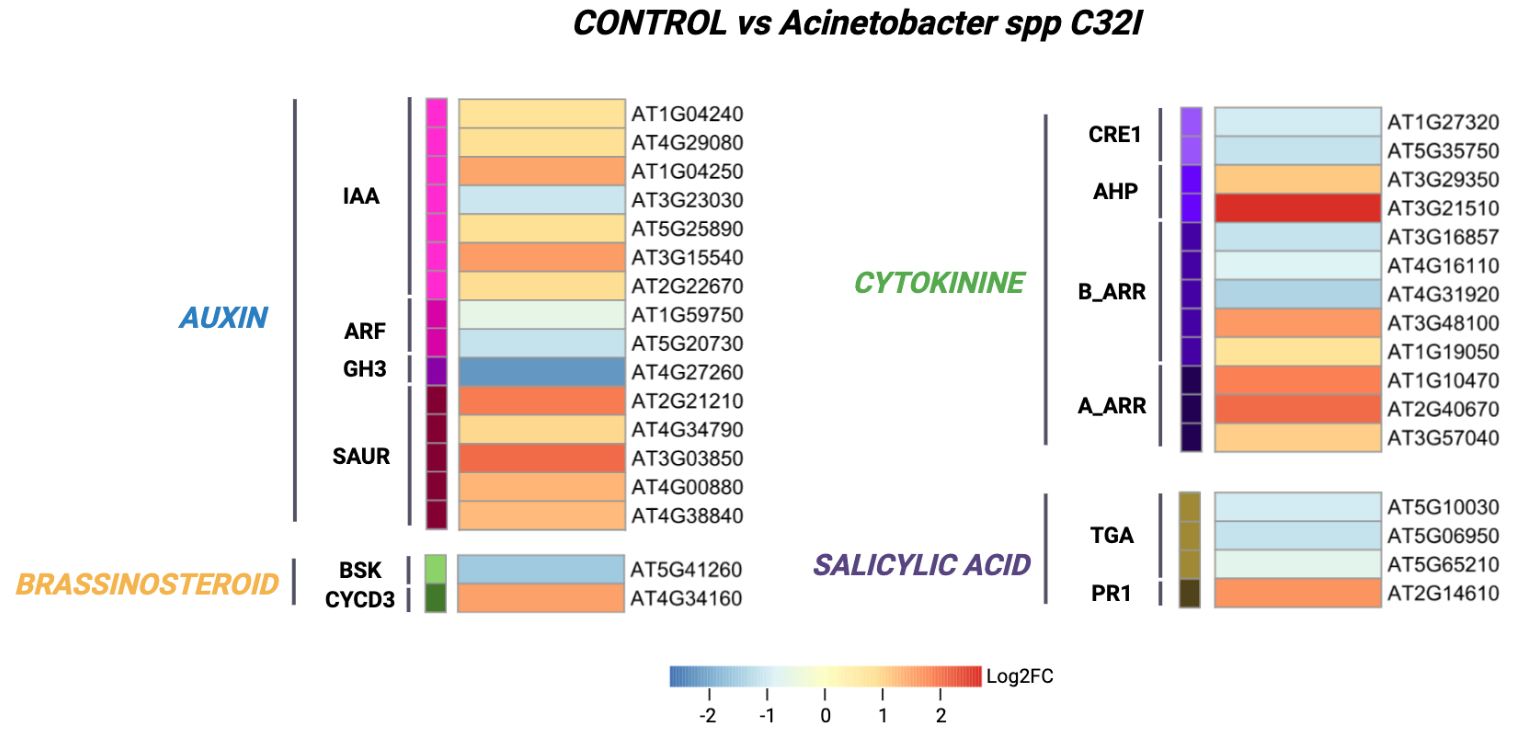

Supplement: Supplementary file 5 — Supplementary file5 (TIFF 217 KB) [file 11103_2024_1444_MOESM5_ESM.tiff]

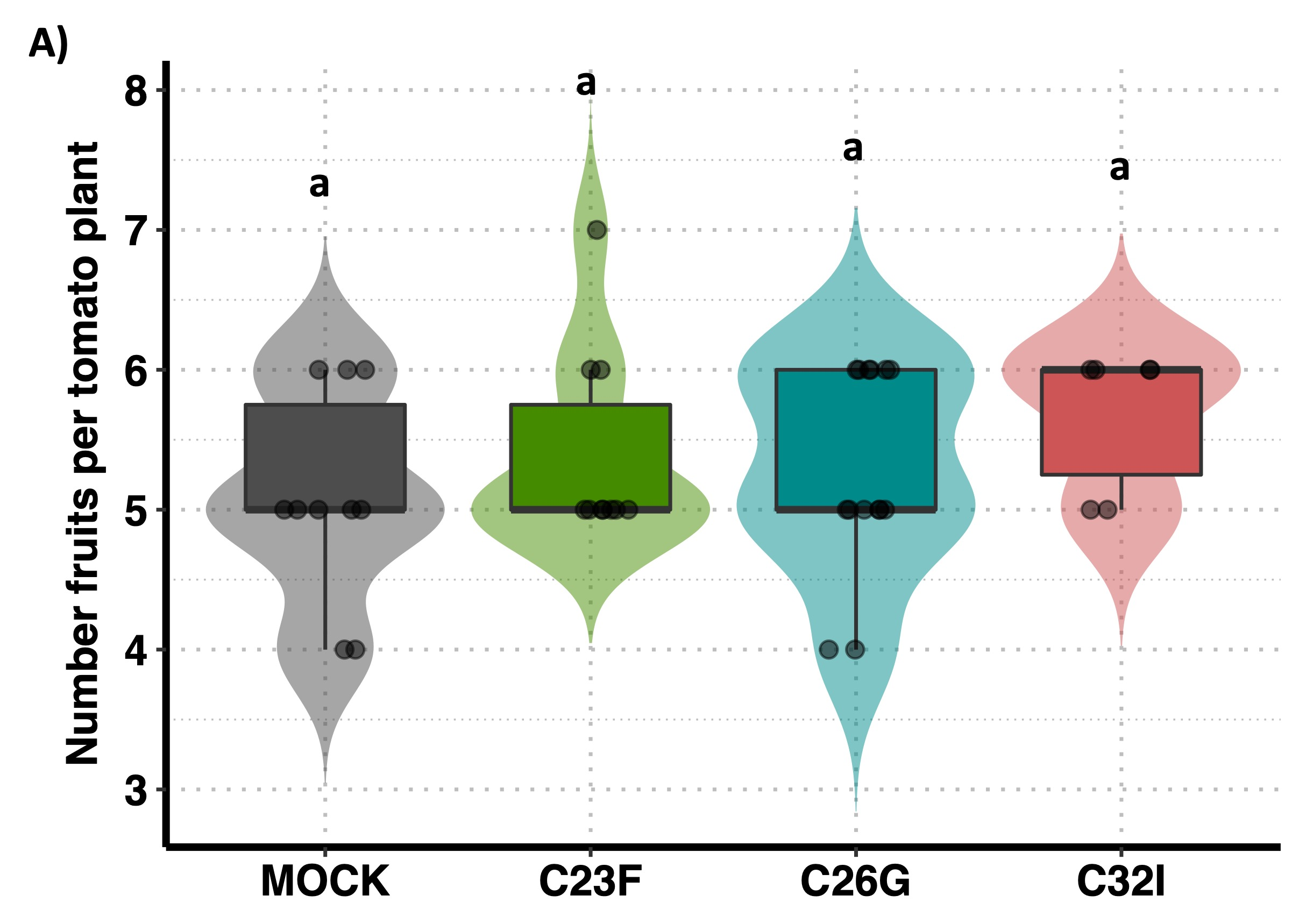

Supplement: Supplementary file 6 — Supplementary file6 (TIFF 12155 KB) [file 11103_2024_1444_MOESM6_ESM.tiff]
